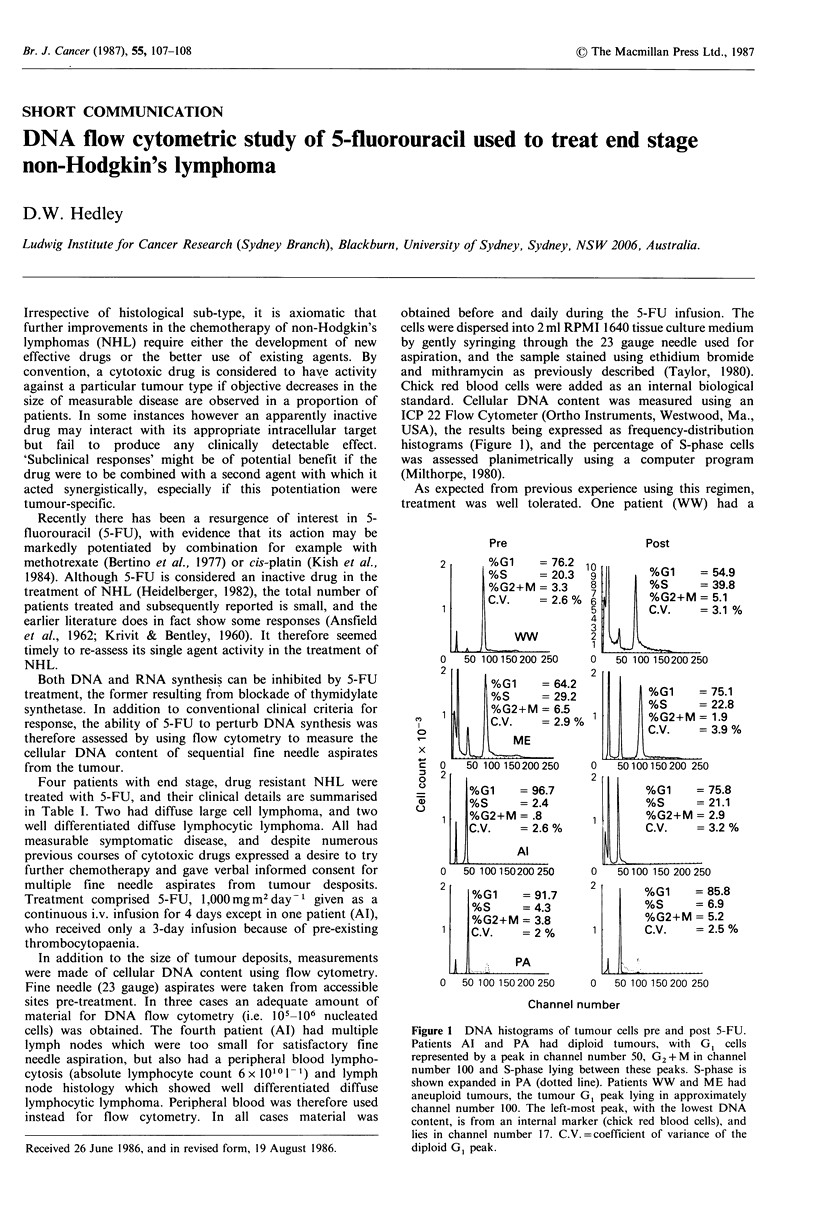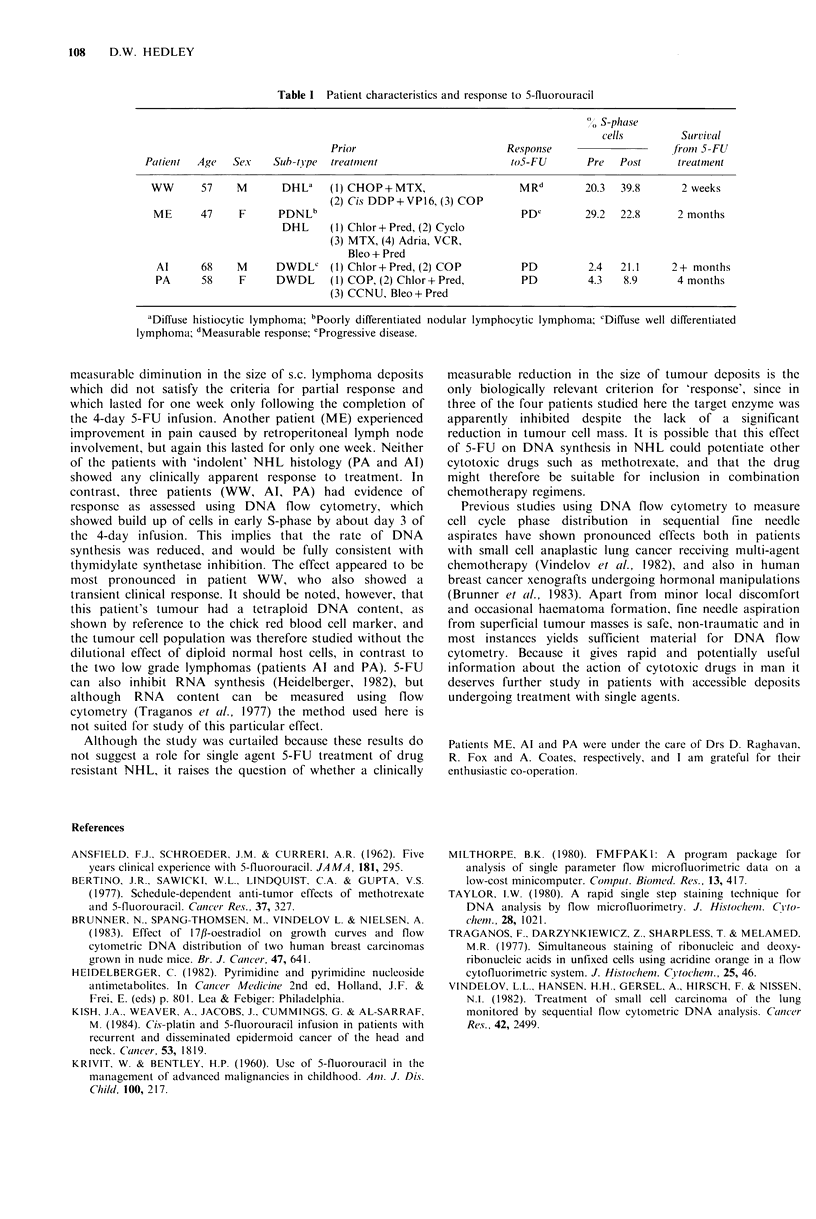# DNA flow cytometric study of 5-fluorouracil used to treat end stage non-Hodgkin's lymphoma.

**DOI:** 10.1038/bjc.1987.22

**Published:** 1987-01

**Authors:** D. W. Hedley


*Br. J. Cancer* (1987), **55**, 107–108 © The Macmillan Press Ltd., 1987

### SHORT COMMUNICATION

### DNA flow cytometric study of 5-fluorouracil used to treat end stage non-Hodgkin's lymphoma

D.W. Hedley

*Ludwig Institute for Cancer Research (Sydney Branch), Blackburn, University of Sydney, Sydney, NSW 2006, Australia.*

Irrespective of histological sub-type, it is axiomatic that further improvements in the chemotherapy of non-Hodgkin's lymphomas (NHL) require either the development of new effective drugs or the better use of existing agents. By convention, a cytotoxic drug is considered to have activity against a particular tumour type if objective decreases in the size of measurable disease are observed in a proportion of patients. In some instances however an apparently inactive drug may interact with its appropriate intracellular target but fail to produce any clinically detectable effect. 'Subclinical responses' might be of potential benefit if the drug were to be combined with a second agent with which it acted synergistically, especially if this potentiation were tumour-specific.

Recently there has been a resurgence of interest in 5-fluorouracil (5-FU), with evidence that its action may be markedly potentiated by combination for example with methotrexate (Bertino *et al.*, 1977) or *cis*-platin (Kish *et al.*, 1984). Although 5-FU is considered an inactive drug in the treatment of NHL (Heidelberger, 1982), the total number of patients treated and subsequently reported is small, and the earlier literature does in fact show some responses (Ansfield *et al.*, 1962; Krivit & Bentley, 1960). It therefore seemed timely to re-assess its single agent activity in the treatment of NHL.

Both DNA and RNA synthesis can be inhibited by 5-FU treatment, the former resulting from blockade of thymidylate synthetase. In addition to conventional clinical criteria for response, the ability of 5-FU to perturb DNA synthesis was therefore assessed by using flow cytometry to measure the cellular DNA content of sequential fine needle aspirates from the tumour.

Four patients with end stage, drug resistant NHL were treated with 5-FU, and their clinical details are summarised in Table I. Two had diffuse large cell lymphoma, and two well differentiated diffuse lymphocytic lymphoma. All had measurable symptomatic disease, and despite numerous previous courses of cytotoxic drugs expressed a desire to try further chemotherapy and gave verbal informed consent for multiple fine needle aspirates from tumour desposits. Treatment comprised 5-FU, 1,000 mg m$^2$ day$^{-1}$ given as a continuous i.v. infusion for 4 days except in one patient (AI), who received only a 3-day infusion because of pre-existing thrombocytopaenia.

In addition to the size of tumour deposits, measurements were made of cellular DNA content using flow cytometry. Fine needle (23 gauge) aspirates were taken from accessible sites pre-treatment. In three cases an adequate amount of material for DNA flow cytometry (i.e. $10^5$–$10^6$ nucleated cells) was obtained. The fourth patient (AI) had multiple lymph nodes which were too small for satisfactory fine needle aspiration, but also had a peripheral blood lymphocytosis (absolute lymphocyte count $6\times10^{10}\,l^{-1}$) and lymph node histology which showed well differentiated diffuse lymphocytic lymphoma. Peripheral blood was therefore used instead for flow cytometry. In all cases material was obtained before and daily during the 5-FU infusion. The cells were dispersed into 2 ml RPMI 1640 tissue culture medium by gently syringing through the 23 gauge needle used for aspiration, and the sample stained using ethidium bromide and mithramycin as previously described (Taylor, 1980). Chick red blood cells were added as an internal biological standard. Cellular DNA content was measured using an ICP 22 Flow Cytometer (Ortho Instruments, Westwood, Ma., USA), the results being expressed as frequency-distribution histograms (Figure 1), and the percentage of S-phase cells was assessed planimetrically using a computer program (Milthorpe, 1980).

As expected from previous experience using this regimen, treatment was well tolerated. One patient (WW) had a

**Figure 1** DNA histograms of tumour cells pre and post 5-FU. Patients AI and PA had diploid tumours, with $G_1$ cells represented by a peak in channel number 50, $G_2+M$ in channel number 100 and S-phase lying between these peaks. S-phase is shown expanded in PA (dotted line). Patients WW and ME had aneuploid tumours, the tumour $G_1$ peak lying in approximately channel number 100. The left-most peak, with the lowest DNA content, is from an internal marker (chick red blood cells), and lies in channel number 17. C.V. = coefficient of variance of the diploid $G_1$ peak.

Received 26 June 1986, and in revised form, 19 August 1986.

**Table I** Patient characteristics and response to 5-fluorouracil

| Patient | Age | Sex | Sub-type | Prior treatment | Response to5-FU | % S-phase cells Pre | Post | Survival from 5-FU treatment |
|---|---|---|---|---|---|---|---|---|
| WW | 57 | M | DHL[a] | (1) CHOP+MTX, (2) *Cis* DDP+VP16, (3) COP | MR[d] | 20.3 | 39.8 | 2 weeks |
| ME | 47 | F | PDNL[b] DHL | (1) Chlor+Pred, (2) Cyclo (3) MTX, (4) Adria, VCR, Bleo+Pred | PD[e] | 29.2 | 22.8 | 2 months |
| AI | 68 | M | DWDL[c] | (1) Chlor+Pred, (2) COP | PD | 2.4 | 21.1 | 2+ months |
| PA | 58 | F | DWDL | (1) COP, (2) Chlor+Pred, (3) CCNU, Bleo+Pred | PD | 4.3 | 8.9 | 4 months |

[a]Diffuse histiocytic lymphoma; [b]Poorly differentiated nodular lymphocytic lymphoma; [c]Diffuse well differentiated lymphoma; [d]Measurable response; [e]Progressive disease.

measurable diminution in the size of s.c. lymphoma deposits which did not satisfy the criteria for partial response and which lasted for one week only following the completion of the 4-day 5-FU infusion. Another patient (ME) experienced improvement in pain caused by retroperitoneal lymph node involvement, but again this lasted for only one week. Neither of the patients with 'indolent' NHL histology (PA and AI) showed any clinically apparent response to treatment. In contrast, three patients (WW, AI, PA) had evidence of response as assessed using DNA flow cytometry, which showed build up of cells in early S-phase by about day 3 of the 4-day infusion. This implies that the rate of DNA synthesis was reduced, and would be fully consistent with thymidylate synthetase inhibition. The effect appeared to be most pronounced in patient WW, who also showed a transient clinical response. It should be noted, however, that this patient's tumour had a tetraploid DNA content, as shown by reference to the chick red blood cell marker, and the tumour cell population was therefore studied without the dilutional effect of diploid normal host cells, in contrast to the two low grade lymphomas (patients AI and PA). 5-FU can also inhibit RNA synthesis (Heidelberger, 1982), but although RNA content can be measured using flow cytometry (Traganos *et al.*, 1977) the method used here is not suited for study of this particular effect.

Although the study was curtailed because these results do not suggest a role for single agent 5-FU treatment of drug resistant NHL, it raises the question of whether a clinically measurable reduction in the size of tumour deposits is the only biologically relevant criterion for 'response', since in three of the four patients studied here the target enzyme was apparently inhibited despite the lack of a significant reduction in tumour cell mass. It is possible that this effect of 5-FU on DNA synthesis in NHL could potentiate other cytotoxic drugs such as methotrexate, and that the drug might therefore be suitable for inclusion in combination chemotherapy regimens.

Previous studies using DNA flow cytometry to measure cell cycle phase distribution in sequential fine needle aspirates have shown pronounced effects both in patients with small cell anaplastic lung cancer receiving multi-agent chemotherapy (Vindelov *et al.*, 1982), and also in human breast cancer xenografts undergoing hormonal manipulations (Brunner *et al.*, 1983). Apart from minor local discomfort and occasional haematoma formation, fine needle aspiration from superficial tumour masses is safe, non-traumatic and in most instances yields sufficient material for DNA flow cytometry. Because it gives rapid and potentially useful information about the action of cytotoxic drugs in man it deserves further study in patients with accessible deposits undergoing treatment with single agents.

Patients ME, AI and PA were under the care of Drs D. Raghavan, R. Fox and A. Coates, respectively, and I am grateful for their enthusiastic co-operation.

**References**

ANSFIELD, F.J., SCHROEDER, J.M. & CURRERI, A.R. (1962). Five years clinical experience with 5-fluorouracil. *JAMA*, **181**, 295.

BERTINO, J.R., SAWICKI, W.L., LINDQUIST, C.A. & GUPTA, V.S. (1977). Schedule-dependent anti-tumor effects of methotrexate and 5-fluorouracil. *Cancer Res.*, **37**, 327.

BRUNNER, N., SPANG-THOMSEN, M., VINDELOV L. & NIELSEN, A. (1983). Effect of 17$\beta$-oestradiol on growth curves and flow cytometric DNA distribution of two human breast carcinomas grown in nude mice. *Br. J. Cancer*, **47**, 641.

HEIDELBERGER, C. (1982). Pyrimidine and pyrimidine nucleoside antimetabolites. In *Cancer Medicine* 2nd ed, Holland, J.F. & Frei, E. (eds) p. 801. Lea & Febiger: Philadelphia.

KISH, J.A., WEAVER, A., JACOBS, J., CUMMINGS, G. & AL-SARRAF, M. (1984). *Cis*-platin and 5-fluorouracil infusion in patients with recurrent and disseminated epidermoid cancer of the head and neck. *Cancer*, **53**, 1819.

KRIVIT, W. & BENTLEY, H.P. (1960). Use of 5-fluorouracil in the management of advanced malignancies in childhood. *Am. J. Dis. Child*, **100**, 217.

MILTHORPE, B.K. (1980). FMFPAK1: A program package for analysis of single parameter flow microfluorimetric data on a low-cost minicomputer. *Comput. Biomed. Res.*, **13**, 417.

TAYLOR, I.W. (1980). A rapid single step staining technique for DNA analysis by flow microfluorimetry. *J. Histochem. Cytochem.*, **28**, 1021.

TRAGANOS, F., DARZYNKIEWICZ, Z., SHARPLESS, T. & MELAMED, M.R. (1977). Simultaneous staining of ribonucleic and deoxyribonucleic acids in unfixed cells using acridine orange in a flow cytofluorimetric system. *J. Histochem. Cytochem.*, **25**, 46.

VINDELOV, L.L., HANSEN, H.H., GERSEL, A., HIRSCH, F. & NISSEN, N.I. (1982). Treatment of small cell carcinoma of the lung monitored by sequential flow cytometric DNA analysis. *Cancer Res.*, **42**, 2499.